# Physicochemical and functional properties of the protein–starch interaction in Chinese yam

**DOI:** 10.1002/fsn3.3189

**Published:** 2023-01-08

**Authors:** Yelin Shao, Ruize Jiao, Yingyin Wu, Fangcheng Xu, Yan Li, Qiaojun Jiang, Liang Zhang, Linchun Mao

**Affiliations:** ^1^ College of Biosystems Engineering and Food Science, Zhejiang Key Laboratory of Agro‐Food Processing, Key Laboratory of Agro‐Products Postharvest Handling of Ministry of Agriculture and Rural Affairs Zhejiang University Hangzhou China; ^2^ Department of Agriculture and Biotechnology Wenzhou Vocational College of Science and Technology Wenzhou China; ^3^ Wencheng Institution of Modern Agriculture and Healthcare Industry Wenzhou China; ^4^ Ningbo Research Institute Zhejiang University Ningbo China

**Keywords:** Chinese yam, digestive properties, physicochemical properties, protein, starch

## Abstract

Protein–starch interaction has an important impact on the properties of starchy foods rich in protein, but the contribution of the interaction to Chinese yam still remains unclear. This study aimed to characterize the physicochemical and functional properties related to the possible interaction between starch and protein in Chinese yam. Differential scanning calorimetry and rapid viscosity analyzer results revealed that the gelatinization temperature increased in protein and starch cross‐linked powder, while the peak viscosity and the setback viscosity decreased. The swelling power and solubility at 80°C and 95°C decreased with increasing protein ratio in the powder. In vitro starch digestibility test indicated that a high protein ratio could rapidly reduce digestible starch, but increase both slowly digestible starch and resistant starch. Protein could act as the physical barrier toward starch against heating and digestion to exert the influence on starch properties. Fourier transform infrared spectroscopy test revealed the interaction between protein and starch. These results revealed the role of protein–starch interaction and provided beneficial information for the utilization of Chinese yam.

## Graphical abstract

The study aimed to characterize the interaction between starch and protein and the changes in the physicochemical properties and functional properties of yam.

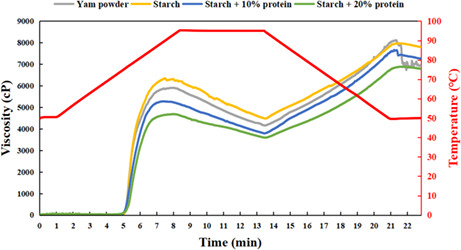

## 1 INTRODUCTION

Starch and protein played important roles in the nutritional quality and processing characteristics of foodstuffs (Zhu et al., 2019). Therefore, the interaction, physicochemical, and digestive properties of starch and protein have become a hot topic of food research. For example, starch–protein powders could be used to formulate new functional foods, including bakery products, snack food, baby food, and desserts, and showed their rich functionality, significant bioactivity, and superior nutritional value (Yang et al., 2019). The interaction between starch and protein has an important influence on the overall texture, stability, and taste of the food, which mainly depends on the thermal properties (Li et al., 2007; Likitwattanasade & Hongsprabhas, 2010). This was mainly because the protein was closely combined with the starch granules or distributed in the gaps of the starch granules by hydrogen bonds, van der Waals forces, and electrostatic, which affected the reaction process of starch in water and heat (Zhu et al., 2019). The interactions of components from different crops have also attracted attention, such as molecule interactions of whey protein with potato starch (Guo et al., 2021; Lin et al., 2022; Liu et al., 2021). In addition, protein could interfere with the starch digestion process by blocking enzyme‐binding sites and ultimately promote starch malabsorption (Oates, 1997). Starch digestibility and glycemic index were affected by protein, which in turn influenced sugar level management strategies and human health (Lal et al., 2021). Therefore, the in‐depth study on starch–protein interaction could help to understand and improve the quality of starch‐based foods.

A subspecies of Chinese yam (*Dioscorea opposita* Thunb.) was recently reported due to its low amylose content and related physicochemical and structural properties (Shao et al., 2020). The high content of protein was expected to play an important role in the nutritional and edible quality of the yam. There have been studies on Chinese yam starch, including native starch, resistant starch, hydrothermally treated starch (Yu et al., 2021; Zou et al., 2020). However, the contribution of protein and its interaction with starch to the physicochemical and digestive properties of the yam still remain unclear.

The purpose of this study was to explore the interaction between starch and protein by confocal laser scanning microscopy and Fourier transform infrared spectroscopy. The physicochemical and functional properties related to the possible interaction between starch and protein were evaluated in terms of gelatinization and pasting properties, solubility, swelling power, and starch digestibility. The information covered in this study would help us to better understand the interaction mechanism between yam starch and protein, and to make effective use of yam resources.

## 2 MATERIALS AND METHODS

### 2.1 Materials

Tubers of Chinese yam (Wennuo No.1) were obtained from Wencheng County, Wenzhou City, Zhejiang, China in October 2019.

### 2.2 Yam powder preparation

Yam powder was prepared referring to Xie et al. (2011). The yam tubers were cut and mixed with deionized water (1:2) in a blender (FL1928; Fuling Technology Co., Ltd), stirred at a speed of 15,294 *g* for 2 min, and then dried in a fume hood (40°C) for 48 h. Protein and starch content of the yam powder was 14.0% and 78.0%, respectively.

### 2.3 Starch extraction

The method of our previous study (Shao et al., 2020) was applied. Cut tubers were homogenized with the same weight of deionized water in the blender. The slurry was filtered through a 200‐mesh sieve with the residue washed twice with distilled water and the filtrate stood for 2 h. Then, the supernatant was decanted, and the precipitated starch layer was resuspended with deionized water. After repeating eight times, the starch was resuspended in ethyl alcohol and dried at 45°C for 24 h (about 10% moisture), and collected by filtration through a 100‐mesh sieve.

### 2.4 Protein isolation

The isolation of yam protein was referred to Hu et al. (2018). Tubers were washed, cut, peeled, and mixed with distilled water (pH 9.0). After stirring at 20°C for 30 min, the mixture was centrifuged at 2655 *g* for 20 min. Then, the supernatant was filtered through a double‐layer cloth using 2 M HCl and magnetically stirred for 1 h. The slurry was then centrifuged at 5000 rpm for 30 min at 20°C. The precipitate was dissolved in distilled water, with pH adjusted to 7.0, then ultrafiltered and lyophilized to prepare the protein.

### 2.5 Mixture of starch and protein

Protein was mixed with starch at various ratios of 0%, 10%, and 20% (w/w), and the mixtures were evenly vibrated and shaken. For subsequent tests, the yam powder or mixtures were stirred in water for 10 min to obtain sufficient dispersion.

### 2.6 Differential scanning calorimetry (DSC)

The gelatinization properties of samples were analyzed by Mettler DSC 1 professional thermal analyzer (ZCEC‐130263F, Mettler‐Toledo, Switzerland) according to Shao et al. (2020). The sample (5 mg, 10% water content) and water (10 mg) were sealed in an aluminum crucible. After equilibration at 4°C for 24 h and then at room temperature for 1 h, the crucible was held at 20°C for 1 min in the DSC furnace and then heated from 20°C to 110°C at a rate of 10°C/min. The data obtained included the onset temperature (*T*_o_), the peak temperature (*T*_p_), the conclusion temperature (*T*_c_), and the crystal melting enthalpy (Δ*H*).

### 2.7 Rapid viscosity analysis (RVA)

A modular compact rheometer (MCR 302, Anton Paar, Austria) was used to measure the pasting properties according to Shao et al. (2020). The pasting temperature (PT), peak viscosity (PV), breakdown viscosity (BV), cold paste viscosity (CV), final viscosity (FV), and setback viscosity (SV) were obtained from the profile. SV = FV – CV.

### 2.8 Swelling power and solubility test

The powder slurry (2% w/v) was heated in a shaking water bath at 80°C or 95°C for 30 min and then cooled to room temperature. After centrifugation at 1699 *g* for 15 min, the supernatant was dried at 105°C for 2 h (Shao et al., 2020).

The swelling power and solubility of the powder were calculated as follows:

$$\begin{array}{c} \text{Solubility}\;\left(\mathrm{SOL},\%\right)\\ =\text{Weight of dried supernatant}\times 100/\text{weight of powder} \end{array}$$

$$\begin{array}{c} \text{Swelling power}\;\left(\%\right)\\ =\left(\text{weight of wet sediment}\times 100\right)/\text{weight of powder}\times \left(100\%-\mathrm{SOL}\%\right) \end{array}$$

### 2.9 In vitro starch digestion

The method of Englyst et al. (1992) was applied to digest starch in powders in vitro. Powder (0.5 g) with 5 ml of distilled water was well mixed, stirred, and maintained in 100°C bath for 30 min, then cooled down to 25°C. The mixture was added with 3 ml of 0.5 M HCl‐KCl solution (pH 1.5) which contained 7 mg/ml pepsin and incubated at 37°C for 60 min. Then, the mixture was added with 2 ml of sodium acetate buffer (0.5 M) which contained 2.5 mg/ml amyloglucosidase (Sigma A‐7095) and 10 mg/ml pancreatic amylase (Sigma P7545). Samples were taken at 0, 10, 20, 60, and 120 min after enzyme addition. After quickly inactivating the enzymes in a boiling water bath, the glucose content was measured. The hydrolysis rates of rapidly digestible starch (RDS, digested <20 min), slowly digestible starch (SDS, digested during 20–120 min), and resistant starch (RS, not digested after 120 min) were calculated.

### 2.10 Confocal microscopic characterization

About 500 mg of powders and 5 ml of distilled water were mixed, stirred, and heated in 100°C water bath for 15 min to obtain a paste. Powders were double‐stained and pasted with fluorescent dyes (0.25 g/dl Rhodamine B and 0.01 g/dl FITC, in acetone) in the dark for 5 min. The stained section was covered carefully with a cover glass. Then, fluorescence images were captured with confocal laser scanning microscopy (CLSM; Leica TCS SP8). Two excitation wavelengths of 543 nm helium–neon laser and 488 nm argon–krypton laser were used to reflect the fluorescence of protein and starch, respectively (Nagano et al., 2008). The distribution and cross‐link of protein and starch could be observed from the images.

### 2.11 Fourier transform infrared spectroscopy

Absorbance spectra of samples were recorded on an FTIR spectrometer (Ava tar370; Nicolet). Samples were prepared using 6% (w/w) powder dispersed in KBr pellets. All powders were dried at 105°C for 24 h, then compressed with KBr, and 256 scans were recorded with a resolution of 4 cm^−1^ and frequency from 400 to 4000 cm^−1^ (Shao et al., 2020).

### 2.12 Statistical analysis

Data were expressed as means ± standard deviation of three replications and performed one‐way analysis of variance (ANOVA) with Duncan's multiple range test (SSR) for statistical analysis by SPSS 25.0 statistical software (Statistical Graphics Corp., Princeton, NJ). When the *p* value was <.05, the value was considered significantly different.

## 3 RESULTS AND DISCUSSION

### 3.1 Gelatinization properties

Gelatinization properties with DSC are shown in Table 1. Pure powder gelatinization illustrated an endothermic peak at 72.8°C. With the protein ratio increasing from 0% to 20%, the onset temperature (*T*_o_), peak temperature (*T*_p_), and conclusion temperature (*T*_c_) of all powders had an increasing tendency. The mixture of 20% protein had the highest *T*_*o*_, *T*_*p*_, and *T*_*c*_, which were 73.8, 76.8, and 80.8°C, respectively. However, accompanying the increase in protein content, the gelatinization enthalpy (Δ*H*) of the powder was significantly reduced. The powder with a protein ratio of 20% had the lowest gelatinization enthalpy of 10.0 J/g.

**TABLE 1:** Influence of protein on gelatinization characteristics of yam starch*.

Samples	Gelatinization parameters**			Δ*H* (J/g)
	*T*_o_ (°C)	*T*_p_ (°C)	*T*_c_ (°C)	
Yam powder (14.0% protein)	73.1 ± 0.3^a^	75.8 ± 0.3^a^	79.1 ± 0.8^b^	10.7 ± 0.6^c^
Starch (0.5% protein)	70.2 ± 0.8^b^	72.8 ± 0.7^c^	78.2 ± 0.2^bc^	13.9 ± 0.3^a^
Starch +10% protein	71.0 ± 0.6^b^	73.9 ± 0.8^b^	77.8 ± 0.3^c^	12.5 ± 0.7^b^
Starch + 20% protein	73.8 ± 0.4^a^	76.8 ± 0.4^a^	80.8 ± 0.5^a^	10.0 ± 0.1^c^

* Values are means ± SD. Values with different letters in the same column are significantly different (*p* < .05).

** *T*_o_, *T*_p_, *T*_c_, and ΔH indicate onset temperature, peak temperature, conclusion temperature, and crystal melting enthalpy, respectively.

Starch granules absorbed water and expanded under the action of moisture and heat, which would be accompanied by the leaching of small granules (Li et al., 2014). Protein could become the barrier for starch and delay starch gelatinization. The presence of protein may affect the fluidity of water to starch granules, resulting in fewer starch–water interactions and correspondingly reduced gelatinization enthalpy in the starch–protein system (Yang et al., 2019). In addition, the significant decrease in gelatinization enthalpy could also be related to the low relative concentration of starch in the mixture, which was due to the dilution effect caused by the presence of protein. A single endothermic peak was observed in the powder with a protein ratio of not more than 20% (Aguilera & Rojas, 1996; Carvalho et al., 2007), and the protein ratio affected the gelatinization temperature and enthalpy value.

### 3.2 Pasting properties

**FIGURE 1:**
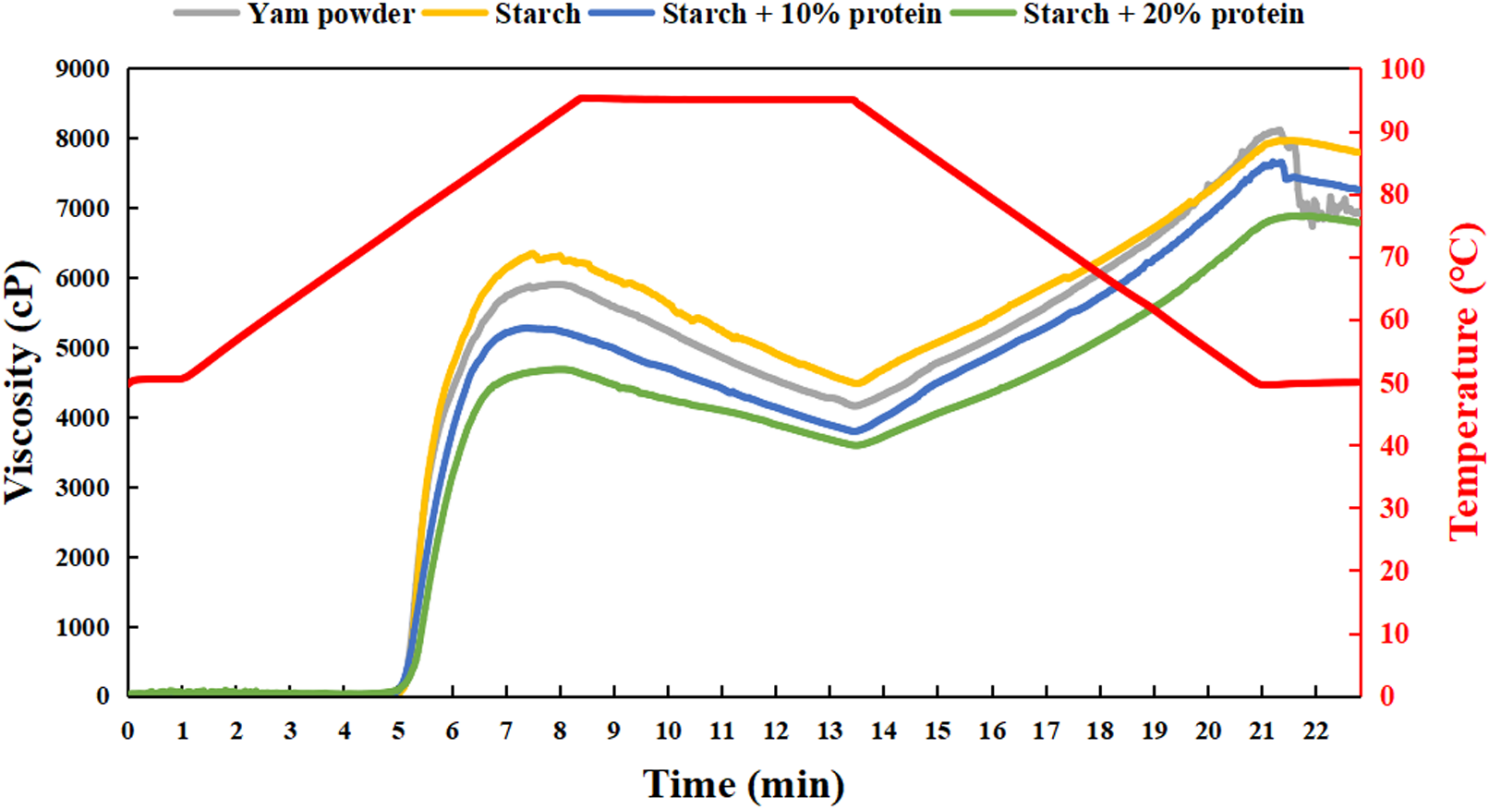
RVA curve of four samples. (a), yam powder (14.0% protein); (b), starch (0.5% protein); (c), starch +10% protein; (d), starch +20% protein.

The pasting properties of starch–protein powders were determined and the results are shown in Table 2 and Figure 1. During the heating process from 50°C to 95°C, the powders of each group began to gelatinize at 73–75°C, then reached the peak viscosity at 80–90°C, and the viscosity rose again after the temperature dropped from 95°C. The pure starch group showed the highest PV, BV, SV, and FV of 6877, 2027, 4129, and 8113 cP, respectively. With the increase of the protein ratio, the peak viscosity of each group had a significant downward trend from 6877 to 4443 cP, but there was no significant difference in their gelatinization temperature.

**TABLE 2:** Influence of protein on pasting properties of yam starch*.

Samples	Pasting parameters**				
	PT (°C)	PV (cP)	BV (cP)	SV (cP)	FV (cP)
Yam powder (14.0% protein)	74.65 ± 0.40^a^	4656 ± 207^c^	1361 ± 301^b^	3278 ± 93^b^	7125 ± 137^b^
Starch (0.5% protein)	74.07 ± 0.09^a^	6877 ± 131^a^	2027 ± 221^a^	4129 ± 174^a^	8113 ± 644^a^
Starch +10% protein	74.57 ± 0.17^a^	5276 ± 107^b^	1379 ± 144^b^	3479 ± 75^b^	7309 ± 57^b^
Starch +20% protein	73.75 ± 2.17^a^	4443 ± 91^c^	1092 ± 208^b^	3267 ± 222^b^	6684 ± 121^b^

* Values are means ± SD. Values with different letters in the same column are significantly different (*p* < .05).

** PT, PV, BV, SV, and FV indicate pasting temperature, peak viscosity, breakdown viscosity, setback viscosity, and final viscosity, respectively.

During the heating process, the starch granules absorbed water and swelled, while some ingredients were leached and dissolved in the solution. Continuous heating and water absorption caused the particles to collapse, completely disorder and crystallinity destruction (Zhu., 2015). However, the protein components in the powders were combined with starch granules or distributed in the gaps of starch granules by hydrogen bonding, electrostatic force, and van der Waals force (Chi et al., 2018). By reducing the contact areas and sites of action between starch and water, protein slowed down the process of water absorption and swelling of starch and even caused the pasting process incomplete. This explained why powders with higher protein ratio had lower peak viscosity. In addition, this also explained the differences in breakdown viscosity and setback viscosity. As important indicators reflecting the characteristics of the paste, the stability was reflected by the breakdown viscosity value, and the gelatinization ability or retrogradation trend was reflected by the setback viscosity (Hoover, 2001). During the cooling phase, protein slowed down the leaching process of amylose, and also hindered the reorganization process through a net‐like structure (Gebre‐Mariam & Schmidt, 1998), and made the final viscosity relatively low. Compared with the starch–protein powders, the pure starch group had a more complete gelatinization process, which increased the stability of the overall structure.

### 3.3 Swelling power and water solubility

Swelling power and solubility at 80°C and 95°C are shown in Table 3. The swelling ability of starch indicates the degree of water absorption of starch granules, and the solubility reflects the content of soluble starch, which is also related to the degree of hydration of starch. Moreover, the degree of this interaction was affected by particle morphology, size, crystal structure, and interference from other substances (Li et al., 2018). Swelling powers of pure starch at 80°C and 95°C were both 11.5%, significantly higher than those of powders with 10% and 20% protein ratio. The solubility of powders also showed a decrease with the protein ratio increased. For instance, the powder with 20% protein ratio had the lowest solubility of 8.8% and 9.1% at 80°C and 95°C, respectively.

**TABLE 3:** Influence of protein on solubility and swelling power of yam starch*.

Samples	80°C		95°C	
	Solubility (%)	Swelling (%)	Solubility (%)	Swelling (%)
Yam powder (14.0% protein)	10.9 ± 0.4^a^	9.5 ± 0.3^b^	9.5 ± 0.8^bc^	10.2 ± 0.4^b^
Starch (0.5% protein)	11.0 ± 1.3^a^	11.5 ± 0.1^a^	11.6 ± 0.9^ab^	11.5 ± 0.1^a^
Starch +10% protein	10.5 ± 0.5^a^	9.6 ± 0.3^b^	9.7 ± 0.6^bc^	10.9 ± 1.0^ab^
Starch +20% protein	8.8 ± 0.1^b^	9.8 ± 0.1^b^	9.1 ± 0.3^c^	10.2 ± 0.4^b^

* Values are means ± SD. Values with different letters in the same column are significantly different (*p* < .05).

The swelling and solubility patterns could provide information about the nature of the associative bonds within starch granules (Perez et al., 2011). For the powders, the stacking arrangement and concentration of starch and protein molecules in the granular structure had a critical impact on the swelling power and solubility of starch (Eliasson, 1986). The degree of interaction between the crystalline and the amorphous starch chain determined the degree of hydration of the starch during the heating process. The presence of protein in the powder limited the swelling of starch granules, which was driven by electrostatic complexation. This could explain the decrease in swelling power of starch with protein ratio increased.

### 3.4 In vitro starch digestibility

For the carbohydrates in the human diet, starch was one of the most important sources, and it also largely affected the overall quality of the diet (Okarter & Liu, 2010). The rate and extent of carbohydrate digestion and absorption in the human body were different, which were important aspects of investigating food quality. By measuring the glucose released during each period of starch hydrolysis, the proportion of nutrient starch digested in each stage could be calculated, thereby showing the overall starch digestion process in vitro. As shown in Table 4, there was a significant difference in the RDS content (56.06%–71.59%) between the pure starch sample and the protein‐containing starch sample (*p* < .05). Compared with pure starch, the RDS content of starch–protein samples decreased significantly, and starch digestion was slowed down in a short time. The increase in SDS content indicated that the suppression of the mitigation effect continued. As the protein content increased, the RS starch fraction steadily increased, which meant that the degree of starch digestion decreased. This may be related to the fact that more protein matrix wrapped the starch network, which limited the efficiency of starch decomposition. The reduction in digestion efficiency was reflected in the simultaneous decline in speed and degree. The protein wrapped starch granules and may become an obstacle to delay enzymatic hydrolysis (Svihus et al., 2005). This result indicated that yam with relatively low protein content was more acceptable because of its more digestible properties, especially for people with starch indigestion.

**TABLE 4:** Nutritional fractions (RDS, SDS, and RS content) of starch–protein mixtures*.

Samples	Nutritional fractions		
	RDS, %	SDS, %	RS, %
Yam powder (14.0% protein)	57.07 ± 0.56^c^	29.42 ± 0.73^a^	13.51 ± 0.60^ab^
Starch (0.5% protein)	71.59 ± 0.81^a^	22.04 ± 1.90^b^	6.37 ± 2.16^c^
Starch +10% protein	61.72 ± 3.41^b^	25.73 ± 5.09^ab^	12.55 ± 1.70^b^
Starch +20% protein	56.06 ± 0.67^c^	27.83 ± 1.55^a^	16.11 ± 0.93^a^

* Values are means ± SD. Values with different letters in the same column are significantly different (*p* < .05).

### 3.5 Confocal microscopic characterization

**FIGURE 2:**
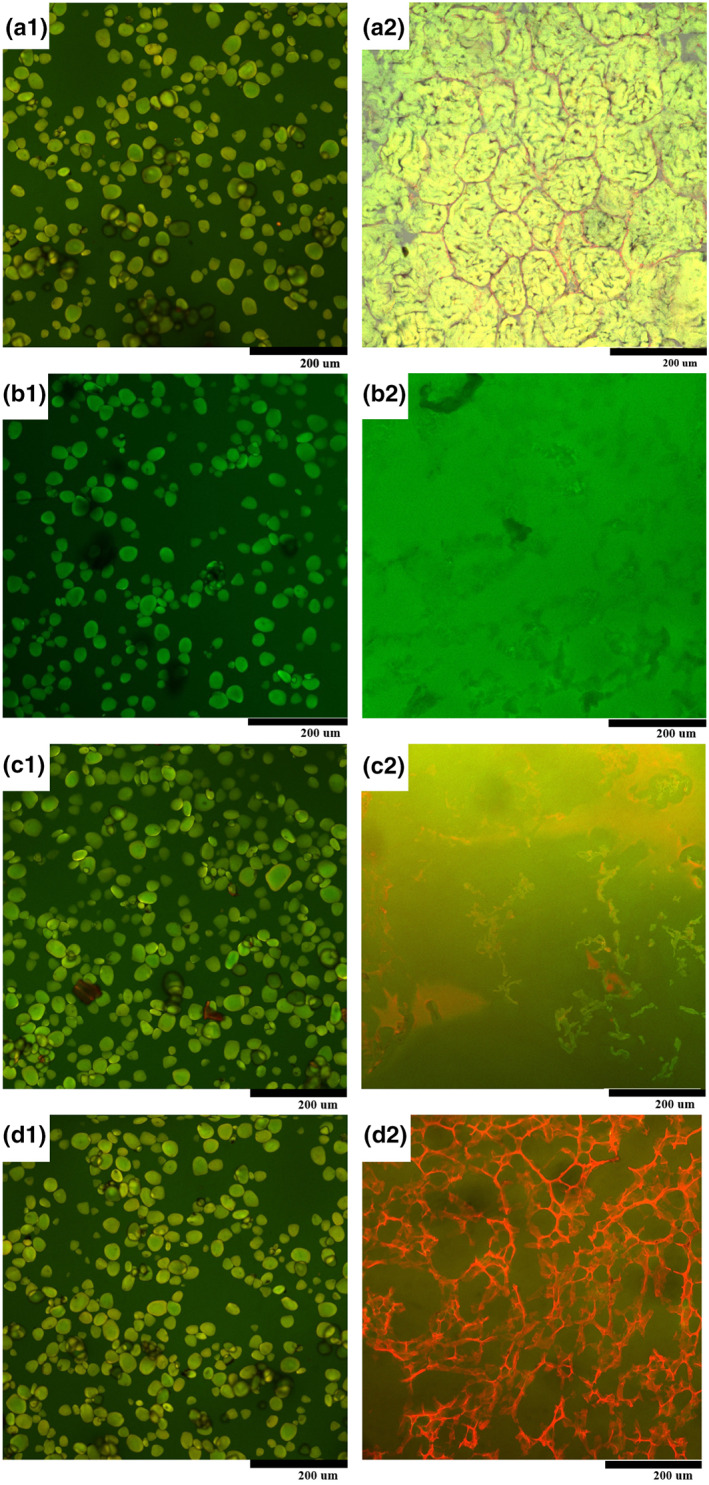
Confocal laser scanning images of the mixture of protein and starch. (a), yam powder (14.0% protein); (b), starch (0.5% protein); (c), starch +10% protein; (d), starch +20% protein. 1, fresh, 2, after heated in 100 °C bath for 15 min. Size bar = 200 μm.

In order to reveal the distribution and structure of the main components in yam, confocal laser scanning microscopy (CLSM) images of fresh and cooked yam powder with different protein–starch ratios are shown in Figure 2. Basically, starch was marked as green by FITC, protein was marked as red by Rhodamine B, and the starch–protein powders at dual wavelengths were shown as yellow under fluorescence.

As shown in Figure 2a1,a2, the green and red distribution areas of fresh and cooked samples basically overlapped. In addition, the fresh yam starch showed independent granular distribution. After cooking, the granules swelled, the volume increased, and the intergranular gaps decreased or even disappeared.

As shown in Figure 2b1,b2, pure starch (green) formed a dense and uniform phase because the starch was gelatinized to the greatest extent. With the addition of yam protein, the uniform structure no longer existed; instead, it was broken into fragmented network structures. Protein absorbed some of the water and heat, expanded the volume through its own heating reaction, and occupied the volume that originally belonged to the starch matrix (Figure 2c1,c2,d1,d2). Because starch was still the main component of the powder, it swelled and cracked during the gelatinization process, the continuous protein matrix was dispersed in the discontinuous starch inclusions.

### 3.6 Short‐range ordered structure

**FIGURE 3:**
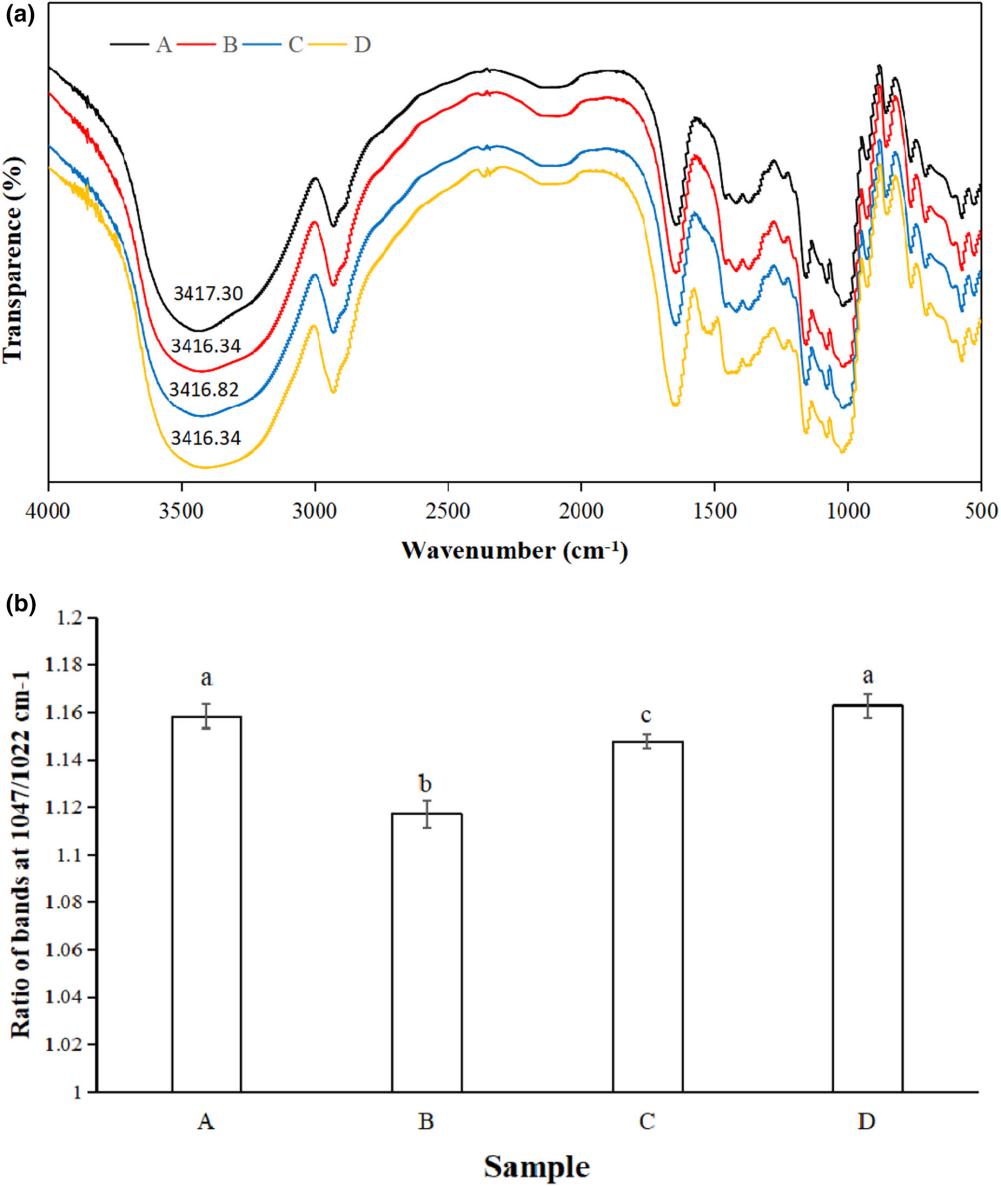
The influence of protein on Fourier infrared structure (a). Band ratio of FTIR spectrum at 1047 and 1022 cm^−1^(b). (a), yam powder (14.0% protein); (b), starch (0.5% protein); (c), starch +10% protein; (d), starch +20% protein.

The FTIR spectrum could show the microstructure of the substance through characteristic peaks. The analysis of yam powders is shown in Figure 3a. The short‐range sequence reflected the double helix sequence. Typical starch peaks at 1047, 1022, and 995 cm^−1^ were related to the short‐range structure and hydrated crystals of starch (vanSoest et al., 1995). The stretch of the glucose ring showed the corresponding band at 930 cm^−1^. For samples containing protein components, the band range above 3000–3600 cm^−1^ was related to intermolecular hydrogen bonding and O‐H stretching vibration. The band at 2930 cm^−1^ was attributed to the C‐H stretching vibration. The typical protein bands at 1655 and 1540 cm^−1^ were assigned to amide I (1580–1720 cm^−1^) and amide II (1480–1580 cm^−1^) (Li et al., 2006). Since only the peaks of starch and protein were observed in the spectrum of this wavelength range, the observation results indicated that there was no covalent interaction between starch and protein.

In order to further study the influence of protein on the short‐range structure of starch, the ratio of 1047/1022 cm^−1^ of powders was evaluated in Figure 3b. It has been confirmed that the absorbance at 1047 and 1022 cm^−1^ was related to the ordered and amorphous structure of starch, and the other band at 995 cm^−1^ was related to hydrated crystals (Sevenou et al., 2002). For the ratio of 1047/1022, yam powder and starch with 20% protein were significantly higher than the other samples, and the pure starch group had the lowest ratio. These results showed that the enhancement of short‐range ordered structure was related to the increase of protein content, that was, protein enhanced the short‐range ordered structure formed by starch to a certain extent.

## 4 CONCLUSIONS

There was a hydrogen bond between protein and starch, mainly due to their interaction, which had a great impact on the physicochemical and digestive properties of the yam starch. Increased protein in the starch could block the process of starch gelatinization and retrogradation with reduced peak viscosity and final viscosity. The swelling power of starch at 80°C and 95°C was decreased, and the water absorption performance was negatively affected by protein. The addition of protein reduced the RDS but increased the SDS and RS. Protein–starch interactions resulted in the formation of a granule mixture, which acted as a physical barrier to starch. With the addition of protein, the short‐range ordered structure of yam powder was significantly enhanced. The physicochemical and functional properties of yam starch were clearly influenced by existing protein.

## CONFLICT OF INTEREST

The authors declare no competing interests.

## Data Availability

The data that support the findings of this study are available on request from the corresponding author. The data are not publicly available due to privacy or ethical restrictions.
